# A Case of Diltiazem-Induced Pustular Rash in an 83-Year-Old Female

**DOI:** 10.1155/2024/9547206

**Published:** 2024-04-02

**Authors:** Ayrton I. Bangolo, Karen Yelton-Torres, Atharv Mahajan, Vrusha Patel, Laiba Sajjad, Prince Ofori Ansong, Monisha Kashyap, Umesh Batura, Madhavi Ravulapalli, Ayodya Perera, Simcha Weissman

**Affiliations:** Department of Internal Medicine, Hackensack University Medical Center, Palisades Medical Center, North Bergen, NJ, USA

## Abstract

Acute generalized exanthematous pustulosis (AGEP) is a rare, acute skin eruption characterized by the development of numerous nonfollicular sterile pustules. Most cases are caused by drug reactions, among which Diltiazem has been incriminated. Herein, we present an 83-year-old female who presented for evaluation of generalized skin rash 3 days after initiation of Diltiazem. She was eventually diagnosed with AGEP, Diltiazem was discontinued, and systemic steroids were administered with the resolution of symptoms. This case report has the objective of encouraging clinicians to include AGEP in the differential diagnosis of skin eruption following the initiation of Diltiazem.

## 1. Introduction

Acute generalized exanthematous pustulosis (AGEP) is a rare drug reaction most often caused by antibiotics [1]. Diltiazem has also been associated with this skin eruption [2]. A rough estimate of the incidence of AGEP is one to five per million per year and can occur at any age [1]. It typically manifests with the rapid development of dozens to hundreds of nonfollicular, sterile, and pinhead-sized pustules on a background of edematous erythema with flexural accentuation [1]. We report the case of an 83-year-old female who presented for evaluation of generalized skin rash with mucosal sparing following initiation of therapy with Diltiazem. A diagnosis of AGEP was made, and the patient was successfully treated with Diltiazem cessation and systemic steroids. With this case report, we hope to raise awareness of this rare condition with Diltiazem use.

## 2. Case Presentation

This is an 83-year-old female with a past medical history significant for atrial fibrillation rate controlled with diltiazem who presented for evaluation of generalized body rash that started 3 days after initiation of Diltiazem therapy. The patient started having macular rash in the elbow flexures which started to progress to the torso and eventually coalesce to form large erythematous maculopapular rash in the torso and inguinal region over the course of 1 week. Of note, the patient was previously on metoprolol for rate control; however, she self-discontinued the medication because it was causing her fatigue. She was then admitted a week prior to encounter for atrial fibrillation with rapid ventricular response, and metoprolol was switched to Diltiazem.

**Figure 1:**
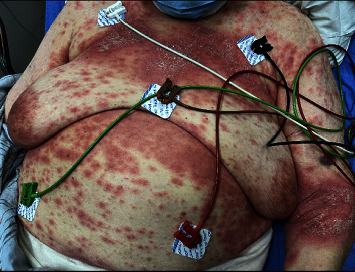
Erythematous macules/papules overlying anterior chest, antecubital fossae, abdomen, inframammary, axillary, and inguinal fold. Typical pinpoint pustules are not seen in this picture.

**Figure 2:**
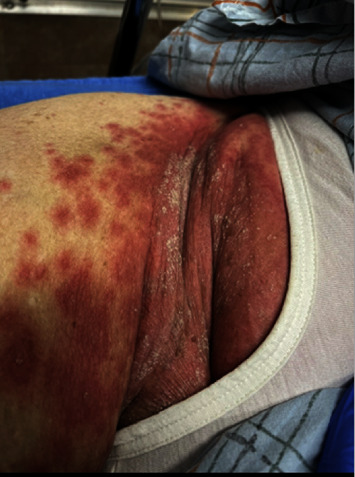
Erythematous macules/papules that appeared to be coalescing into patches/plaques involving the intertriginous area. Typical pustules are not seen in this picture.

Upon arrival in the Emergency Department, she was found to be tachycardic with a temperature of 99.2° F, a heart rate of 157 beats per minute, and hypotensive with a blood pressure of 86/56 millimeter of mercury. She was found to have leukocytosis with white blood cell (WBC) of 33 × 10^3^ (4.0–11.0 × 10^3^). The neutrophil count was 31000 (94%). The physical examination revealed erythematous macules/papules that appeared to be coalescing into patches/plaques overlying the anterior chest, antecubital fossae, abdomen, inframammary, axillary, and inguinal fold. Overlying the erythema was pinpoint pustules and areas of fine-scale/desquamation. These findings can be seen in Figures 1 and 2. The electrocardiogram revealed atrial fibrillation at a rapid rate, and all the infectious work-ups including coxsackie, varicella, parvovirus, rubella, and roseola were negative. Our other differential diagnoses were pustular psoriasis and symmetrical drug-related intertriginous and flexural exanthema (SDRIFE), the age of onset, and the absence of itching without flaky skin, with no personal or family history of psoriasis, making pustular psoriasis unlikely. SDRIFE has been reported with antibiotics (especially amoxicillin), is usually symmetrical, involves the perineal and gluteal regions, or does not have any systemic changes. Our patient had an AGEP validation score of 9 as per the EuroScar study group. A presumptive diagnosis of acute generalized exanthematous pustulosis (AGEP) was made. The patient underwent a skin punch biopsy.

The patient was initiated on amiodarone continuous infusion and high-dose methylprednisolone; Diltiazem was discontinued. The biopsy result showed intraepidermal pustules with papillary edema and neutrophilic infiltrates consistent with AGEP. The patient was eventually transitioned to bisoprolol, and steroids tapered down. The patient's generalized rash started resolving, the rash started to diminish in size, and there was desquamation of the skin with a 2-week taper dose of steroids, and she was discharged from the hospital.

## 3. Discussion

Acute generalized exanthematous pustulosis (AGEP) is an uncommon skin condition primarily triggered by certain medications, carrying a mortality rate of up to 2 percent [3]. Typically, the rash emerges within a few days to weeks following the administration of the implicated drug [3]. Initially manifesting on the face or in skin folds, the eruption swiftly spreads to the torso and limbs, exhibiting either a widespread or localized pattern [4]. In the case of our patient, who had recently initiated Diltiazem therapy known to induce AGEP, the rash appeared three days after commencing the medication. Notably, the eruption initiated in skin folds before spreading to the limbs and torso, while the face and buccal mucosa remained unaffected.

The diagnosis of AGEP relies on clinical and histologic assessments, typically suspected in patients displaying an acute, febrile pustular rash shortly after initiating drug therapy, notably antibiotics or Diltiazem [1]. In the acute phase, typical indicators include fever exceeding 38°C (100.4°F), elevated white blood cell count with neutrophil predominance, and mild eosinophilia [1]. Confirmatory diagnosis and differentiation from other pustular eruptions necessitate histologic examination of a skin biopsy [1]. A characteristic feature of AGEP on histologic evaluation is the presence of spongiform subcorneal and/or intraepidermal pustules [5, 6]. Although our patient did not exhibit fever, significant leukocytosis was noted alongside the pustular rash within days of initiating Diltiazem therapy. Histologic analysis of the skin biopsy confirmed spongiotic dermatitis with pustules, thereby establishing the diagnosis of AGEP.

The approach to managing AGEP involves discontinuation of the causative drug, along with providing supportive care and addressing symptoms such as itching and skin inflammation [7]. The use of topical steroids in AGEP is supported by limited case series and clinical observations of their effectiveness in managing pruritic or inflammatory skin disorders [8]. Systemic corticosteroids have also been employed in the treatment of AGEP [8]. In our patient's case, Diltiazem was stopped, and she was initiated on systemic steroids with a gradual taper over several days. Her symptoms persisted for approximately a week before resolving completely.

## 4. Conclusion

Acute generalized exanthematous pustulosis (AGEP) is a rare skin reaction associated with Diltiazem use. Early recognition of this condition is essential, as discontinuation of the offending agent is the mainstay of management. With this case report, we hope to raise awareness of this rare condition associated with Diltiazem and hope to encourage clinicians to include AGEP in the differential of patients with pustular rash shortly after initiation of Diltiazem [9].

## Data Availability

All data generated or analyzed during this study are available from the corresponding author upon request.
